# Stereolithography Apparatus Evolution: Enhancing Throughput and Efficiency of Pharmaceutical Formulation Development

**DOI:** 10.3390/pharmaceutics13050616

**Published:** 2021-04-25

**Authors:** Carlo Curti, Daniel J. Kirby, Craig A. Russell

**Affiliations:** School of Pharmacy, Aston University, Aston Triangle, Birmingham B4 7ET, UK; curtic@aston.ac.uk (C.C.); D.J.KIRBY1@aston.ac.uk (D.J.K.)

**Keywords:** 3D printing, stereolithography, digital light processing, solid oral dosage forms, formulation development, personalised medicine, cost effectiveness, lean production, sustainability

## Abstract

Pharmaceutical applications of 3D printing technologies are growing rapidly. Among these, vat photopolymerisation (VP) techniques, including Stereolithography (SLA) hold much promise for their potential to deliver personalised medicines on-demand. SLA 3D printing offers advantageous features for pharmaceutical production, such as operating at room temperature and offering an unrivaled printing resolution. However, since conventional SLA apparatus are designed to operate with large volumes of a single photopolymer resin, significant throughput limitations remain. This, coupled with the limited choice of biocompatible polymers and photoinitiators available, hold back the pharmaceutical development using such technologies. Hence, the aim of this work was to develop a novel SLA apparatus specifically designed to allow rapid and efficient screening of pharmaceutical photopolymer formulations. A commercially available SLA apparatus was modified by designing and fabricating a novel resin tank and build platform able to 3D print up to 12 different formulations at a single time, reducing the amount of sample resin required by 20-fold. The novel SLA apparatus was subsequently used to conduct a high throughput screening of 156 placebo photopolymer formulations. The efficiency of the equipment and formulation printability outcomes were evaluated. Improved time and cost efficiency by 91.66% and 94.99%, respectively, has been confirmed using the modified SLA apparatus to deliver high quality, highly printable outputs, thus evidencing that such modifications offer a robust and reliable tool to optimize the throughput and efficiency of vat photopolymerisation techniques in formulation development processes, which can, in turn, support future clinical applications.

## 1. Introduction

Three-dimensional (3D) printing is defined as a set of manufacturing technologies used to make parts by adding material in a layer-by-layer fashion [1]. Due to its appealing features, 3D printing has received great interest from the pharmaceutical field, especially following the 2015 FDA approval of the first 3D-printed drug product, Spritam. Since then, interest has aroused fast and, so far, several 3D-printing technologies have been used, understood, and improved [2], and particular emphasis has been posed on its potential applications in delivering personalised medicine [3]. This particular use has been motivated by the intrinsic flexibility of 3D printers, that are able to fabricate solid oral dosage forms with bespoke properties potentially with no need to alter the formulation [4], in contrast to conventional tableting techniques which are not customizable at reasonable costs and only have limited geometries achievable. For example, the recently FDA-approved T19 rheumatoid arthritis drug, designed as a chronotherapeutic drug delivery system targeting the circadian symptoms of the disease, achieves its particular release profile thanks to the complex inner geometry fabricated through 3D printing [5]. Such an approach would complement the standard mass production of medicines, embracing a highly patient-centric method foreseen to revolutionise pharmacotherapy [6].

Promising 3D printing applications currently rely on Fused Deposition Modelling (FDM), Selective Laser Sintering (SLS) and vat photopolymerisation (VP) techniques [7]. Each of these technologies differ in the way the layers are built; for example, in FDM a drug loaded filament is thermally extruded into the desired geometry, while in SLS thin layers of powdered raw material are sintered by a laser [8]. VP techniques, such as Stereolithography (SLA) and Digital Light Processing (DLP) instead operate through light-induced curing of photosensible resins [8].

Formulation of solid oral dosage forms with different 3D printing methods mainly affect features such as drug release, mechanical properties, and external appearance. FDM and related techniques have been used for a wide range of applications and allowed the manufacturing of variously engineered solid dosage forms [9,10,11,12], while SLS has been investigated for its power to fabricate rapidly disintegrating tablets [13,14,15,16,17]. SLA and DLP 3D printing have been instead used to fabricate controlled release dosage forms, hydrogels and polypills [7,18,19,20,21,22,23].

While FDM currently stands as a frontrunner in the advanced development of 3D-printed solid oral dosage forms, its disadvantages should be considered as, for example, process limitations restrict the number of drugs that can be used due to potential process-induced thermal degradation [24,25]. This is especially true considering that FDM is generally coupled with hot-melt extrusion, thus doubling the incidence of thermal challenge and chance of stability issues [26]. Furthermore, developing drug-loaded filaments with satisfactory mechanical properties for extrusion and 3D printing can be challenging [27]. Similarly, heat-induced degradation could also affect SLS 3D-printed products due to the rise in temperature caused by the sintering activity of the laser [7]. Additionally, the required feed-stock material can suffer from flowability issues, particularly when the powder is thinly spread at the completion of each layer [28].

Such limitations are not shared by VP techniques, as both SLA and DLP do not rely on heat for fabrication and do not require powders. Instead, each layer is manufactured by either a laser beam (SLA) or a digital projector screen (DLP) inducing the polymerisation of a drug-loaded resin. VP is also a very accurate process with high printing resolution, enabling the fabrication of solid oral dosage forms with greater patient acceptability over other techniques such as FDM and SLS [7,29,30].

However, pharmaceutical applications of VP technologies still account for the smaller share and remain underdeveloped [7,31]. This is particularly dependent upon throughput limitations related to the impossibility of printing simultaneously using low volumes of different resins [21], thus making formulation development processes time consuming and cost inefficient. Although some discontinuous methods to overcome this limitation have been suggested [32], the overall process needs to be improved.

Furthermore, limitations are posed by the lack of materials suitable for VP; commercially available photopolymer resins have been designed mainly for engineering purposes, where tough and resistant structures are needed with high crosslinking observed in the polymerised networks [33,34]. However, from a pharmaceutical perspective, such mechanical attributes are not desirable as orally administered dosage forms should completely break down to release their drug content and to then be eliminated with no risk of leaving tablet fragments in the gastrointestinal tract [35]. Additionally, despite the existence of biocompatible commercially available resins designed for special applications, such as dentistry [36,37], only a limited number of photopolymer formulations have been investigated for pharmaceutical applications [20,22,32,38]. Such limitations, therefore, lay the foundations for an extensive screening of photopolymer formulations and their respective evaluation. SLA and DLP 3D printers currently on the market are designed to operate using large volumes of a single resin at any one time [21] allowing for large prints, which can be advantageous in prototyping and similar applications. This is not required or desirable in pharmaceutical formulation development and, consequently, without addressing such aspects, developing novel formulations would require an unnecessarily large amount of resin resulting in a less than economical process.

Hence, the aim of this work was to design and fabricate a novel SLA apparatus able to 3D print solid dosage forms using low volumes and multiple formulations at the same time, with the view to maximising throughput and cost-effectiveness of the technique. Lean production principles of avoiding waste related to ‘inventory’, ‘overproduction’ and ‘waiting’ were followed as a general guideline to identify critical areas to address to improve the technique for pharmaceutical applications [39]. Furthermore, the purpose of developing a novel SLA apparatus arrangement was to employ a high throughput screening of novel pharmaceutical photopolymer resins to address the lack of formulations for VP technologies. Screened formulations were evaluated based on their printability outcomes with the view to develop a pool of multi-purpose, drug-loadable resins that can be flexibly used to deliver safe, effective, and personalised dosage forms.

## 2. Materials

Clear resin V4.0 (Formlabs Inc., Somerville, MA, USA) was purchased as a commercially available photopolymer for SLA 3D printing. Clear silicon glue was purchased from Loctite (Henkel Corp., Düsseldorf, Germany). Aluminium tape was acquired from 3M, USA. Propan-2-ol was obtained from Fisher Scientific, Loughborough, UK. A 150 (width) × 150 (depth) × 100 (height) mm H30-6082-T6 aluminium alloy block was purchased from John Keatley Metals (KeatleyMetals Ltd., Birmingham, UK). Polyethylene glycol diacrylate (PEGDA–MW 250, 575 and 700) and N-vinyl-pyrrolidone were used as reactive oligomers and monomer, respectively. Diphenyl 2,4,6-trimethyl benzoyl phosphine oxide (TPO) was used as photoinitiator at a concentration of 1%, 0.5%, 0.1% and 0.05% (*w/w*). Polyethylene glycol 300 (PEG 300), propylene glycol and glycerol were selected as liquid non-reactive fillers at a concentration of 12.5%, 25% and 50% (*w/w*). All chemical reagents were purchased from Sigma-Aldrich, Gillingham, UK.

## 3. Methods

### 3.1. Stereolithography Apparatus

A Form 2 SLA 3D printer (Formlabs Inc., Somerville, MA, USA) was used as a desktop stereolithography apparatus to manufacture all the formulations presented in this work. The Form 2 3D printer is equipped with a 405 nm laser and has a build volume of 145 (width) × 145 (depth) × 175 (height) mm. The feedstock material consists of a photopolymer resin contained in a 200 mL vat. Printed objects are formed on a build platform made of aluminium and plastic, with a build area of 21,025 mm^2^ and a weight of 635.18 g.

### 3.2. Design and 3D Printing of a Modified Build Platform Prototype and Resin Tank

An attachment consisting of twelve compartments to be inserted onto the original resin tank was designed on TinkerCAD (Autodesk Inc., San Rafael, CA, USA). In contrast to the original 200 mL resin tank, each compartment was designed to contain 10 mL of photopolymer resin. To match the novel resin tank, a modified version of the build platform featuring twelve build spots (_12_BP) was also designed using TinkerCAD. Each spot has a build area of 400 mm^2^, allowing the fabrication of single tablets up to 20 mm in diameter. The modified build platform and the resin tank insert were 3D printed with the Form 2 using Clear resin photopolymer; each print was setup using PreForm 2.20.0–Beta 1 (Formlabs Inc., Somerville, MA, USA). The 3D printing of the build platform required 401.38 mL of photopolymer resin and took 31 h and 1 min to be completed. The resin tank insert required 156.33 mL of photopolymer resin and was completed in 9 h and 52 min. Both the modified parts were 3D printed at a resolution (layer thickness) of 100 µm. Following the 3D printing process, each part was placed in propan-2-ol and cleaned in a sonic bath for 20 min to remove any uncured resin. All the necessary supports were removed after drying for 10 min at room temperature. The twelve 3D printed compartments were finally fixed to the silicone layer on the original resin tank using silicone glue, while each spot of the 3D printed _12_BP was covered with 75 µm thick aluminium tape with an adhesion strength of 12N/cm to allow easy removal of printed tablets.

### 3.3. Design and Fabrication of An Aluminium Multi-Build Platform

A twelve spots aluminium build platform (aluminium _12_BP) was designed using SolidWorks (Dassault Systèmes, Vélizy-Villacoublay, France), based on the design of the 3D printed prototype, manufactured through computer numerical control (CNC) milling and finally bead blasted to provide a rough finishing aimed to increase objects’ adherence while printing and to facilitate their release once fabricated; the support fixing the build platform to the SLA apparatus was designed using TinkerCAD and 3D printed with clear resin photopolymer.

### 3.4. Tablet Uniformity Testing

The original build platform (BP), the 3D printed _12_BP and the aluminium _12_BP were connected to the SLA apparatus and used to fabricate cylindrical tablets to evaluate the influence of different build platforms on tablet uniformity. Three batches of twelve tablets each were 3D printed on each platform. All tablets manufactured at this stage were composed of Clear Resin photopolymer V4.0 (Formlabs Inc., Somerville, MA, USA). After 3D printing, ten tablets per batch were randomly picked to carry out tablet uniformity tests. Measurements were taken for tablet weight, thickness and diameter. Tablets were designed using TinkerCAD. A conventional cylindrical geometry with a diameter of 12.0 mm and a thickness of 4.0 mm was selected and tablets were printed both directly on the build platform and oriented to 45° using printing supports to evaluate the impact of scaffolds on tablet uniformity. Tablet thickness and diameter were measured using a digital caliper; tablet weight was measured on a precision balance. Statistical analyses were performed using SPSS Version 26.0.0.0 (IBM Corp., Armonk, NY, USA).

### 3.5. Formulation of Photopolymer Resins and 3D Printing

Cylindrical tablet CAD files were uploaded as stereolithographic files (.stl) using PreForm 2.20.0–Beta 1 and set to be printed directly on the build platform. In total, 156 photopolymer formulations were designed based on different combinations of PEGDA 250, PEGDA 575, PEGDA 700, N-vinyl-pyrrolidone, PEG 300, glycerol, propylene glycol and TPO, as described in Table S1. Then, 10 mL of each formulation was prepared by mixing the liquid photopolymers and eventual fillers with the powdered photoinitiator, and stirred for 12 h or until complete dissolution of all the ingredients and were kept away from light sources. Then, twelve formulations per time were loaded in the novel resin tank for 3D printing. Each run took 4.37 h to be completed at a resolution of 25 µm, 1.95 h at 50 µm and 1.1 h at 100 µm.

### 3.6. Printability Evaluation

**Figure 1:**
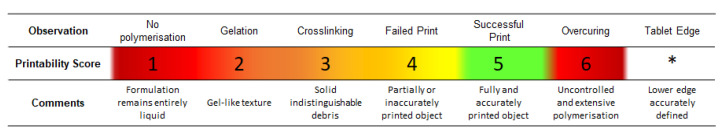
Printability scale designed to evaluate photopolymer formulations’ printability outcomes.

Photopolymer formulations’ printability outcomes were evaluated according to a six-point arbitrary scale (Figure 1). A printability score (PS) from 1 to 6 was assigned to each formulation based on visual inspection. An extra score was assigned to formulations providing 3D printed tablets with a well-defined lower edge. This was introduced to differentiate between formulations showing overcuring only in the first layers rather than the whole tablet. Inclusion criteria were then based on formulations reaching a printability score of 5 and/or showing a defined edge (*) after printing a cylindrical test tablet.

### 3.7. Novel SLA Apparatus Cost-Effectiveness

The total time required to screen 156 formulations using the novel SLA apparatus, as well as the volume of formulation samples needed and the cost per each formulation prepared, was noted. A cost-effectiveness comparison between the two SLA apparatus was carried out by calculating the time required to screen one formulation at a time using the original apparatus and the costs for preparing 200 mL of each photopolymer formulation as required by the original capacity resin tank of 200 mL.

### 3.8. Resin Recovery Efficiency Evaluation

**Figure 2:**
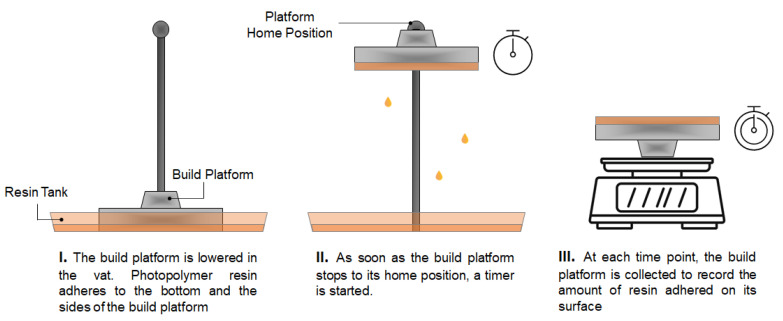
Schematic diagram of the method developed to evaluate photopolymer resin wastage due to adherence on the build platform at the completion of a print.

The original BP and the aluminium _12_BP were weighed separately. Each platform was then connected to the 3D printer and a print was initiated. Once the platform was completely lowered in the resin tank and covered in photopolymer resin, the print was aborted to allow the BP to home. As soon as the initial position was reached, a timer was started and the platform collected to be weighed again at given timepoints (Figure 2). The experimental procedure was carried out at room temperature. The volume of resin adhered to the build platform at each timepoint was calculated using Equation (1):

V_n_ = (w_n_ − w_i_)/ρ (1)

where V_n_ indicates the volume of resin adhering to the build platform at the n-time point, w_n_ is the weight of the build platform at the n-time, w_i_ is the initial weight of the build platform and ρ is the resin relative density.

The economic loss relative to the wasted resin at the n-timepoint was calculated using Equation (2):

Economic loss (£) = [(w_n_ − w_i_)/ρ] ⨯ resin cost (£/mL) (2)

## 4. Results and Discussion

### 4.1. Stereolithography Apparatus Evolution

**Figure 3:**
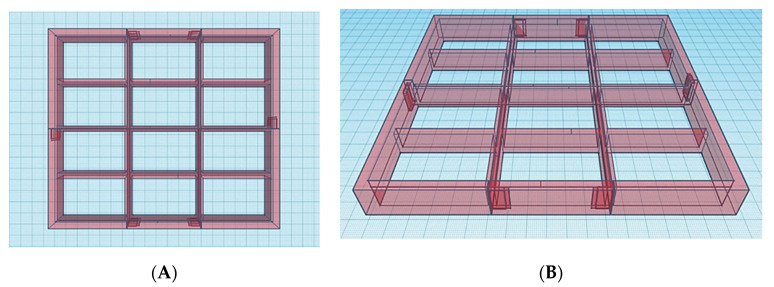
(**A**) Top and (**B**) front view of the CAD model designed for the resin tank insert. Each square on the blue background corresponds to a surface area of 1 cm^2^.

**Figure 4:**
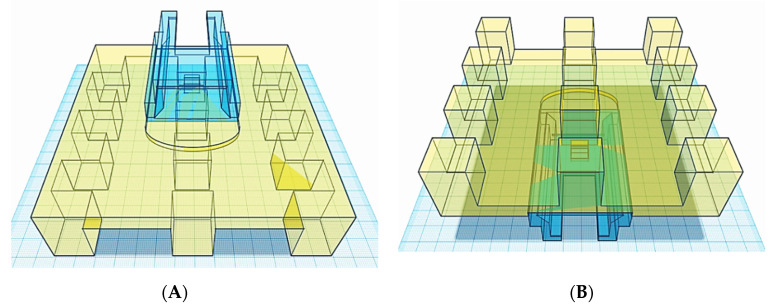
(**A**) Top and (**B**) bottom view of the CAD model used for the twelve-spots build platform (_12_BP). Each square on the blue background corresponds to a surface area of 1 cm^2^.

In order to address throughput limitations of conventional vat polymerisation apparatus equipped with a single, large-volume resin tank, the first step in modifying the commercial SLA apparatus was the design of twelve resin compartments and a build platform featuring twelve separate build areas (_12_BP) (Figure 3 and Figure 4). The dimensions were selected to be the minimum dimensions to allow for tablet printing and resin-depth changes upon submersion of the printing platform.

**Figure 5:**
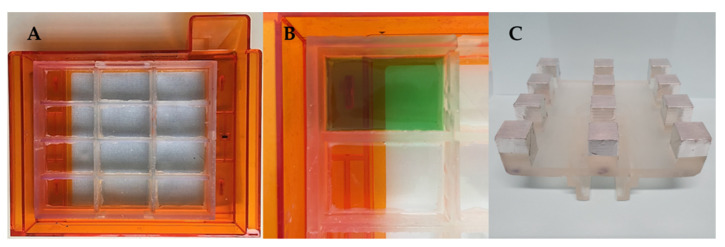
(**A**) Twelve-vats 3D printed insert placed and fixed onto the silicon layer of the original resin tank, (**B**) compartments’ sealing test, and (**C**) bottom view of the 3D printed _12_BP showing aluminium tape used to coat each build spot.

The CAD files for the novel components were then sent to the 3D printer to be manufactured. The resin tank inserts were fixed onto the original resin tank and tested for being watertight by alternately filling the compartments with a green-coloured solution and leaving them overnight to assess any leaks from the filled compartment to the next ones (Figure 5A,B), while the 3D printed _12_BP was covered with aluminium tape to allow for ease of removal of printed dosage forms (Figure 5C).

**Figure 6:**
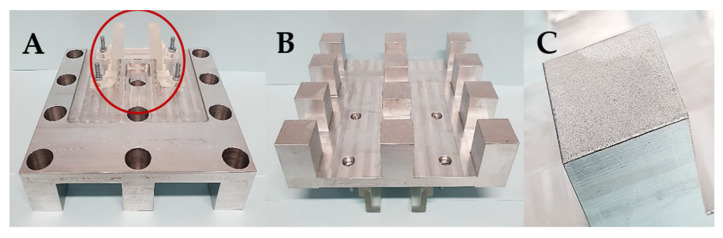
(**A**) Top and (**B**) bottom view of the aluminium _12_BP fabricated through CNC milling. The novel design includes hollow build-spots, thus reducing the weight of the platform. A 3D printed attachment (circled in red) allows firm attachment of the platform to the SLA apparatus. (**C**) Detail of bead-blasted surface of the aluminium _12_BP. Such a finish was selected to optimise the adhesion of objects during the 3D printing process and to facilitate their release at the end of the print.

Subsequently, after the 3D printed _12_BP was shown to be firmly connected to the printer and compatible with the novel twelve-vats resin tank, a final version of the build platform made of aluminium (aluminium _12_BP) was fabricated through CNC milling and fixed to the SLA apparatus using a 3D printed joint, which was easily replaceable in the case of breakage (Figure 6). Aluminium was selected due to its similarity to the original component and its density of 2.70 g/cm^3^ [40]. The fully assembled aluminium _12_BP final weight was 625.15 g, resulting in a 1.58% decrease in weight compared to the original BP. Such weight was estimated before manufacturing and maintained by drilling holes in the aluminium block (visible in Figure 6A) to obtain a finished product whose weight could not damage the moving parts of the SLA apparatus.

**Figure 7:**
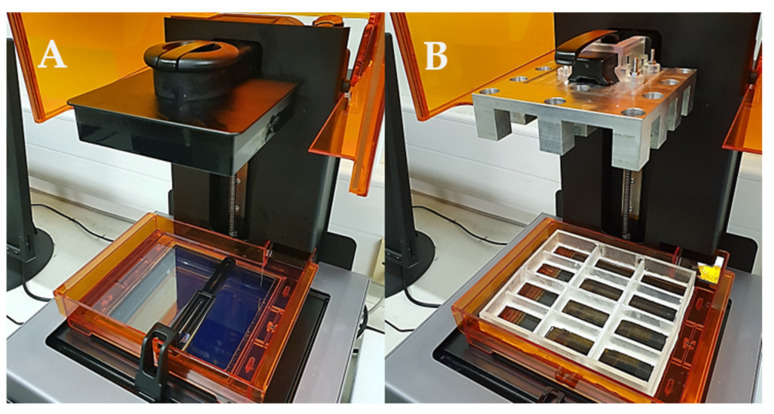
Comparison of (**A**) the original Form 2 SLA apparatus and (**B**) the novel SLA apparatus developed. The modified SLA apparatus allows operation with up to twelve different photopolymer formulations simultaneously. Each vat has been designed to contain 10 mL of resin formulation.

With the novel resin tank and build platform in place, a commercial stereolithography apparatus was converted into a piece of equipment able to print multiple formulations at a single time with a fraction of the material originally required (Figure 7). Such novel apparatus was designed with the intention to conduct the high-throughput screening of photopolymer formulations aimed to identify printable candidates to produce solid oral dosage forms.

**Figure 8:**
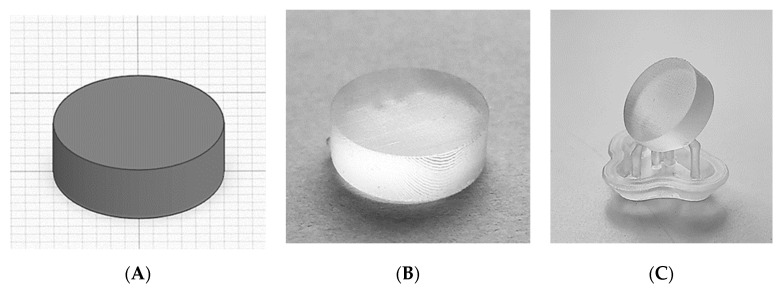
(**A**) CAD model and (**B**) 3D printed cylindrical tablet printed directly on the build platform. (**C**) Tablet 3D printed using supports.

The modified apparatus’ reliability was assessed by printing cylindrical tablets using a commercially available resin photopolymer. Twelve tablets were printed on the aluminium _12_BP, with and without supports (Figure 8). The printability score (PS) assigned to both the types of fabricated tablets was 5*, indicating a successful print with accurately defined edges in all cases.

### 4.2. Tablet Uniformity Testing

**Figure 9:**
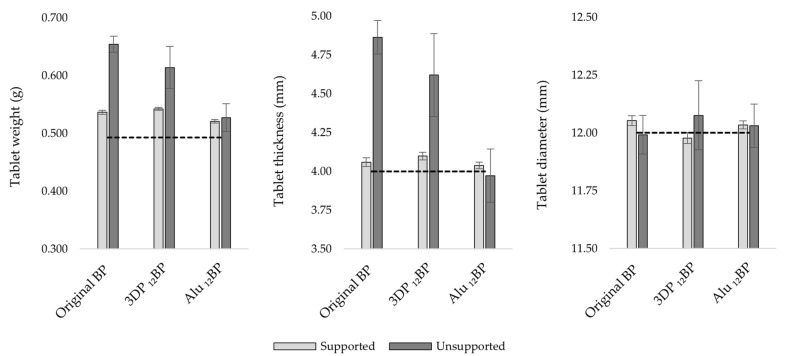
Tablet uniformity of weight (**left**), thickness (**middle**), and diameter (**right**). Black and white bars represent data from tablets printed with and without supports, respectively. Black dashed lines indicate the theoretical value for each parameter. Results were expressed as average (*n* = 30). Error bars indicate standard deviation.

Three batches of twelve tablets each were fabricated using the original BP, the 3D printed _12_BP and the aluminium _12_BP. Each batch was 3D printed with and without supports to evaluate their impact on tablet uniformity. Results for the uniformity of weight, thickness and diameter are shown in Figure 9.

Considering a theoretical value for tablet weight of 0.493 g, estimated from tablet volume and resin density, the percent relative error (%E_r_) calculated for the original BP, the 3D printed _12_BP and the aluminium _12_BP was 32.67%, 24.50% and 6.90%, respectively, for tablets printed directly on the build platform, while the relative standard deviation (RSD) was 2.15%, 5.91% and 4.56%, respectively. However, the introduction of printing supports resulted in the fabrication of more accurate and precise batches, as shown by a decrease in the %E_r_ and RSD, respectively, to 8.81% and 0.61% (original BP), 10.05% and 0.46% (3D printed _12_BP), 5.64% and 0.61% (aluminium BP_12_).

A similar trend was observed when evaluating tablet thickness; when comparing the original BP and the 3D-printed _12_BP, there was a %E_r_ of 21.57% and 15.52%, with an RSD of 2.22% and 5.77%, respectively, when printing without supports. As per tablet weight, introducing printing scaffolds lowered the %E_r_ and the RSD, respectively, to 1.46% and 0.69% (original BP), 2.44% and 0.62% (3D printed _12_BP). However, tablets printed using the aluminium _12_BP showed a %E_r_ and RSD, respectively, of 0.94% and 0.54% (with supports), −0.71% and 4.34% (without supports), indicating better uniformity performances when the aluminium _12_BP was used.

A more uniform pattern was observed when evaluating tablet diameter. In fact, the %E_r_ observed considering a theoretical diameter of 12 mm was 0.44% (RSD = 0.18%), -0.19% (RSD = 0.20%) and 0.29% (RSD = 0.15%) with the original BP, the 3D printed _12_BP and the aluminium _12_BP, respectively, when printing with supports. Printing tablets directly on the build platform led instead to a %E_r_ of −0.07% (RSD = 0.70%), 0.63% (RSD = 1.23%) and 0.26% (RSD = 0.78%) with respect of the original BP, the 3D printed _12_BP and the aluminium _12_BP.

A multivariate analysis of variance (MANOVA), coupled with a Tukey post hoc test, was performed to evaluate the effect of the build platform used evidenced a statistically significant difference (*p* < 0.05) in tablet weight, thickness and diameter when the 3D printed _12_BP was compared to the original BP and tablets were printed directly on the BP. Comparing weight and thickness uniformity results of unsupported tablets fabricated with the aluminium _12_BP and the original BP also resulted in a statistically significant difference (*p* < 0.05), while no difference (*p* > 0.05) was observed for tablet diameter.

**Figure 10:**
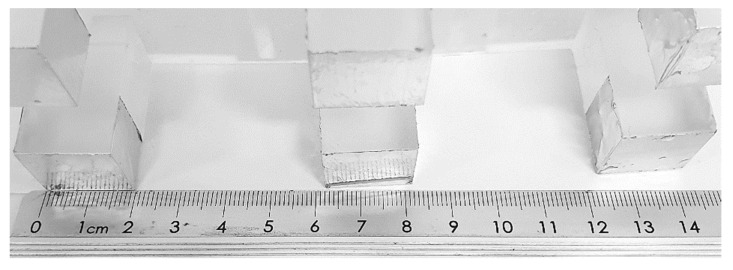
Bending of the 3D printed _12_BP leading to misalignment of the BP in the SLA apparatus.

The results firstly suggest that tablet thickness is the most susceptible factor to inhomogeneity; since it is generally observed that the tablet thickness is higher than the expected value, it is likely that this also led to a gain in weight and, therefore, inhomogeneity in tablet weight uniformity. In particular, high differences were related to the use of the 3D-printed _12_BP. A potential explanation can be found in the loss of structural integrity observed in the 3D printed _12_BP over time (Figure 10). The clear resin photopolymer used to manufacture the 3D printed _12_BP suffers, in fact, from significant limitations in terms of mechanical properties and tends to deform over time and light exposure [41,42,43]. Even a minimal change in the BP geometry could eventually result in a print with poor dimension accuracy. As aluminium does not share such a limitation, this would explain the significant improvements in tablet uniformity when the aluminium _12_BP was used.

Secondly, it was found that introducing printing supports considerably improved tablet uniformity when using the original BP and the 3D printed _12_BP. In comparison with the original BP, no statistically significant difference (*p* > 0.05) in weight and thickness uniformity was observed for tablets fabricated on the 3D printed _12_BP. Supported tablets printed on the aluminium _12_BP also showed no significant difference in terms of uniformity of thickness and diameter when compared to results obtained from tablets produced on the original BP. Such improvements are compatible with the general recommendation to use printing supports for fabricating objects with minimum risk of size inaccuracies [44].

However, it should be considered that printing scaffolds require extra material to be fabricated and are a primary source of waste (Table 1).

### 4.3. Resin Recovery Efficiency Evaluation

**Figure 11:**
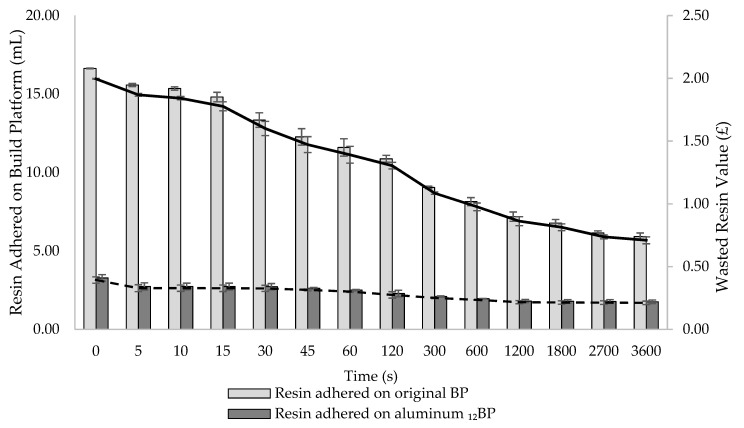
Comparison of photopolymer resin waste generated by adherence onto the original BP and the aluminum _12_BP over a period of one hour. The straight curve indicates the economic loss using the original SLA apparatus. The dashed curve indicates the economic loss using the modified SLA apparatus. Error bars indicate standard deviation of the measurements (*n* = 3).

At the completion of each print, the BP is automatically lifted and later removed by an operator to collect the fabricated dosage forms, while any uncured resin remaining on the platform is removed and disposed of. Attempts to manually recover resin adhered to the BP using metal tools could result in accidentally recovering partially cured resin debris, or in scratching the aluminium surface with risk to contaminate the feedstock material. Although manual removal determines most of the final resin loss, the amount of material wasted, and its related cost, have not been defined before. As a variable amount of recoverable resin drops from the BP into the resin tank as soon as a print is finished, it was hypothesized that the time the platform was left in the 3D printer before being removed was a critical parameter to estimate the final material wastage. In fact, the longer the BP remains connected to the SLA apparatus, the more photopolymer resin is recovered and saved. Therefore, the impact of the time the BP is left in the 3D printer after a print is completed on the amount of resin eventually wasted was investigated (Figure 11). Both the original SLA apparatus and its modified version were compared to assess potential differences in their capacity to generate time-dependent resin waste. Cost implications of such waste generation were also assessed.

Measurements were taken at 14 time points covering a period of 1 h. At t = 0 s, 16.63 mL of resin adhered to the original BP, while only 3.28 mL were recorded on the aluminum _12_BP. At t = 3600 s, the amount of adhered material was quantified as 5.92 and 1.76 mL for the original BP and the aluminium _12_BP, respectively.

According to the results, it can be stated that, if the BP is left in the SLA apparatus at the end of a print for an increasing amount of time, a clear effect on reducing resin waste is observed. Furthermore, the aluminium _12_BP used in the novel SLA apparatus has proven to reduce the amount of adhering resin by 70.27%, in comparison to the original BP; avoiding such waste would allow for the saving of enough material to produce an additional 11 and 3 tablets (based on a 0.5 mL tablet volume) using the original and the modified SLA apparatus, respectively.

From a cost point of view, the effect of time on material saving, as well as differences between the use of the original and the modified SLA apparatus, are evident (Figure 11). The economic loss due to the resin adhering on the build platforms just returned in position after a print (t = 0 s) was quantified as GBP 2.00 for the original SLA apparatus versus GBP 0.39 for the modified version. By leaving the platform above the tank until the end of the experiment (t = 3600 s), wasted resin value decreased to GBP 0.71 and GBP 0.21 for the original and the modified build platforms, respectively. It should be noted that the suggested model was based on the use of a commercial photopolymer resin not intended for pharmaceuticals applications. The lack of commercially available resins designed for pharmaceutical manufacturing necessitates the on-site production of photopolymer formulations consisting of polymers, photoinitiators, active pharmaceutical ingredients and other excipients, which eventually increase the final cost per mL. For example, considering the highest cost per mL for the formulations discussed in this work (Table S1), and assuming comparable materials’ behaviour, GBP 4.16 worth of photopolymer resin would be wasted at t = 0s using the original SLA apparatus, while the resin loss using the modified build platform would be quantified as GBP 0.82 at the same timepoint. Recovering photopolymer resin from the build platforms for one hour would instead decrease the value of wasted material to GBP 1.48 and GBP 0.44 using the original and the modified SLA apparatus, respectively.

Ultimately, our findings aim to suggest a potential solution to minimise photopolymer resin wastage by avoiding the immediate removal of the build platform after the completion of dosage forms of 3D printing. This would, in fact, allow a certain amount of resin to be time-dependently recovered and reused, with no need of operator intervention. While the effect of time and the type of BP used have been evaluated, other factors, such as photopolymer resins’ viscosity and surface tension, should also be investigated, in order to establish a solid model to universally predict material wastage and identify the amount of time providing the highest recovery.

In fact, it is likely that the production of personalised dosage forms in clinical settings, such as hospital pharmacies, will have higher costs than the mass production of drugs at an industrial level, and it is, therefore, necessary to maximise process cost-effectiveness [45].

### 4.4. High-Throughput Screening of Pharmaceutical Photopolymer Formulations

#### 4.4.1. Novel SLA Apparatus Cost-Effectiveness Evaluation

The modified SLA apparatus was used to carry out a printability screening of 156 pharmaceutical photopolymer formulations. The total time required for the screening, formulations amount needed, and the related costs are reported in Table 2. A comparison of the same parameters estimated considering the use of the original apparatus is also delineated.

The developed modified SLA apparatus proved to dramatically reduce both the time and the sample amount required to conduct systematic screening of photopolymer formulations. In particular, the use of the novel SLA apparatus resulted in a 91.66% reduction in the amount of time needed to complete the screening, and 95% less raw materials being used.

These results make the introduction of the modified apparatus into formulation development processes a promising tool to enhance the application of SLA 3D printing in pharmaceutics, which has been limited until now. Furthermore, our aim was to bridge the gap between general use SLA equipment and those designed for research applications, with the view of developing SLA 3D printers specifically designed for pharmaceutical purposes in the future.

#### 4.4.2. Printability Outcomes Evaluation

**Figure 12:**
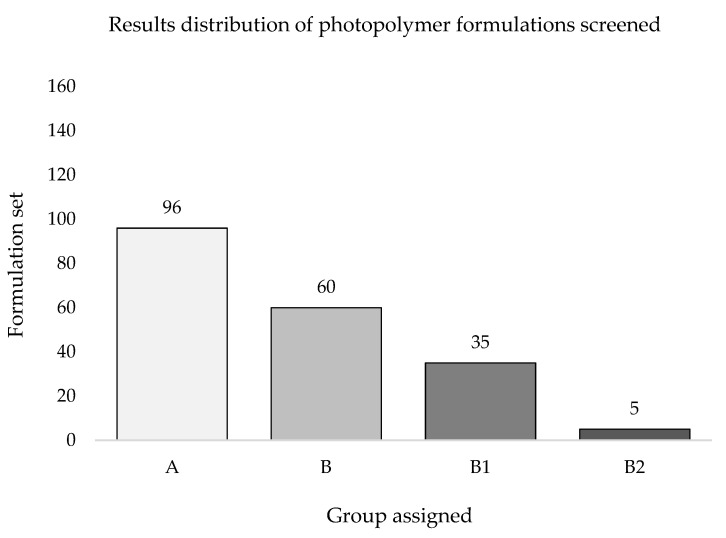
Group classification of photopolymer formulations screened. Group A indicates formulations with PS≠5 (*n* = 96); group B reports formulations with a PS = 5 or PS = * at least at one printing resolution (*n* = 60); groups B1 (*n* = 35) and B2 (*n* = 5) list formulations with a PS = 5* at least at one or at each printing resolution used, respectively.

Based on the inclusion criteria, the whole set of photopolymer formulations screened was classified in four groups (Figure 12). Out of the 156 formulations tested, 96 provided a PS≠5 indicating poor printability outcomes (Figure 12, group A), while the remaining 60 formulations met the eligibility criteria by reaching a PS = 5 or showing defined edges (*) with at least one printing resolution, making up a pool labelled as Printable Formulations (PF, *n* = 60) (Figure 12, group B).

Formulations included in group B were then subclassified into groups B1 (*n* = 35; formulations reaching PS = 5* at least for one printing resolution) and B2 (*n* = 5; formulations reaching PS = 5* at each printing resolution). Formulations belonging to groups B1 and B2 were jointly labelled as Best Formulations (BF, *n* = 40).

A detailed table, including the composition of each formulation, the printability score assigned at each resolution, and the group to which it belongs, is shown in Table S1.

**Figure 13:**
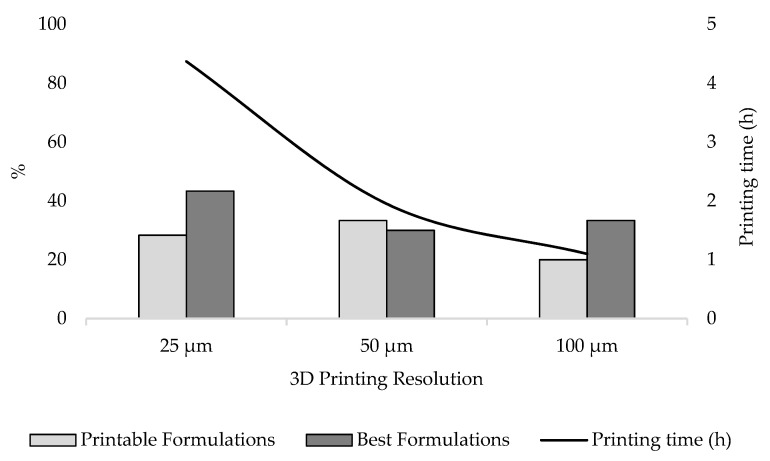
3D printing resolution effect on printability outcomes. Printable Formulations and Best Formulations are expressed as a percentage of Group B (*n* = 60). The black line indicates the printing time required to complete a 12-formulations screening.

The effect of 3D printing resolution on printability outcomes was also investigated (Figure 13). Selecting a resolution of 25 µm resulted in 43.3% of group B formulations being classified as BF, followed by 30.0% and 33.3% selecting a resolution of 50 and 100 µm, respectively. In total, 33.3% of group B formulations were instead classified as PF when screened at 50 µm, while a reduction to 28.3% and 20.0% was observed when printing at 25 and 100 µm, respectively. Overall, of all the formulations classified in group B, 71.7% met targeted printability criteria using a printing resolution of 25 µm, whereas a decrease in printing resolution to 50 and 100 µm also reduced the fraction of formulations providing satisfactory outcomes to 63.3% and 53.3%, respectively.

It should, however, be considered that printing with a resolution of 25 µm increases printing time by 55.38% and 74.83% compared to using a layer thickness of 50 and 100 µm, respectively. Despite the better results observed using higher resolution, the increase in production time should not be underestimated. The implementation of SLA 3D printing in clinical settings to produce personalised dosage forms will in fact be possible if the overall efficiency of the process is optimised, reducing costs and production times, and ensuring the safety and efficacy of the printed medicines [45,46]. It is, therefore, essential to identify novel formulations, designed to provide best printability even at low resolution.

Our systematic screening has shown how modifying a commercial SLA apparatus allows us to address the limitation of identifying printable resin formulations with a significant reduction both in terms of time and costs. Furthermore, the application of the modified SLA apparatus in a clinical scenario would allow for the printing of multiple formulations at the same time to provide patients with their personalised medicines in reasonable time.

## 5. Conclusions

A commercial SLA apparatus was modified into a novel, multimaterial device specifically designed to address the limitations of SLA 3D printing in pharmaceutical applications. The novel SLA apparatus was tested by carrying out a high-throughput screening to identify pharmaceutical photopolymer formulations with satisfactory printability and was proved to considerably reduce the time and economic resources needed. Furthermore, potential areas of wastage were identified and solutions to address them were described with the view to enhance SLA 3D printing feasibility at a clinical level. In conclusion, the novel apparatus’ power to 3D print different formulations at the same time may not only be advantageous at a formulation development stage, but also in clinical scenarios where different solid oral dosage forms can be produced together using the same 3D printer, making access to personalised medicines to patients more achievable.

## Figures and Tables

**Table 1:** Weight of 3D-printed tablets and relative supports produced using the original BP and the 3D-printed _12_BP. Measurements were taken before and after supports were removed from tablets (*n* = 10). Material waste percentage is expressed as the ratio of support weight over initial weight.

Material Waste Assessment	Original BP	3D Printed _12_BP
Initial weight (g)	16.096	16.280
Supports weight (g)	12.544	16.701
Material waste percentage (%)	43.799	50.638

**Table 2:** Cost-effectiveness comparison of the two SLA apparatus used to screen 156 pharmaceutical photopolymer formulations.

Apparatus Used	Time (h)	Sample Required (L)	Materials Cost (GBP)
Modified SLA Apparatus	96.42	1.56	292.21
Original SLA Apparatus	1157.00	31.20	5844.19

## Data Availability

The data presented in this study are contained within this article.

## Supplementary Materials

The following are available online at https://www.mdpi.com/article/10.3390/pharmaceutics13050616/s1, Table S1: % *w/w* composition of the 156 photopolymer formulations prepared and screened. Printability score assigned per formulation at each printing resolution tested and the classification group of each formulation are reported. Costs/mL per formulation are described in the right column.
