# Supplementary material for: Stereolithography Apparatus Evolution: Enhancing Throughput and Efficiency of Pharmaceutical Formulation Development
Source: Pharmaceutics. 2021 Apr 25;13(5):616. doi: 10.3390/pharmaceutics13050616 (PMC8145482; doi:10.3390/pharmaceutics13050616)
Supplement: Supplementary file 1 [file pharmaceutics-13-00616-s001.zip › pharmaceutics-1182498 sup with modified footer .pdf]

pharmaceutics

Supplementary Materials: Stereolithography Apparatus Evolution: Enhancing Throughput and Efficiency of Pharmaceutical Formulation Development

Carlo Curti, Daniel J. Kirby and Craig A. Russell *

Citation: Curti, C.; Hussain, T.; Kirby, D.J.; Russell, C.A. Stereolithography Apparatus Evolution: Enhancing Throughput and Efficiency of Pharmaceutical Formulation Development. *Pharmaceutics* **2021**, *13*, 616. https://doi.org/10.3390/pharmaceutics13050616

Academic Editor: Natalja Genina

Received: 29 March 2021
Accepted: 21 April 2021
Published: 12 May 2021

Publisher's Note: MDPI stays neutral with regard to jurisdictional claims in published maps and institutional affiliations.

Copyright: © 2021 by the author. Licensee MDPI, Basel, Switzerland. This article is an open access article distributed under the terms and conditions of the Creative Commons Attribution (CC BY) license (http://creativecommons.org/licenses/by/4.0/).

Table S1. % *w/w* composition of the 156 photopolymer formulations prepared and screened. Printability score assigned per formulation at each tested printing resolution and the classification group of each formulation are reported. Costs/mL per formulation are described in the right column.

	PEGDA 250	PEGDA 575	PEGDA 700	PEG 300	Propylene Glycol	Glycerol	N-Vynil Pyrrolidon e	TPO	PS @ 25 μm	PS @ 50 μm	PS @ 100 μm	Group assigned	Cost (£/mL)
**F1**	99.00	-	-	-	-	-	-	1.00	5*	5*	6	B1	0.25
**F2**	99.50	-	-	-	-	-	-	0.50	5*	5*	6	B1	0.23
**F3**	99.90	-	-	-	-	-	-	0.10	4	5	5*	B1	0.21
**F4**	99.95	-	-	-	-	-	-	0.05	4	4	4	A	0.21
**F5**	-	99.00	-	-	-	-	-	1.00	6	6	6	A	0.24
**F6**	-	99.50	-	-	-	-	-	0.50	6	6	6	A	0.22
**F7**	-	99.90	-	-	-	-	-	0.10	5 *	6 *	6 *	B1	0.20
**F8**	-	99.95	-	-	-	-	-	0.05	4	6	6	A	0.20
**F9**	-	-	99.00	-	-	-	-	1.00	6	6	6	A	0.24
**F10**	-	-	99.50	-	-	-	-	0.50	6	6	6	A	0.22
**F11**	-	-	99.90	-	-	-	-	0.10	5*	6*	6	B1	0.20
**F12**	-	-	99.95	-	-	-	-	0.05	5*	6*	5*	B1	0.20
**F13**	86.50	-	-	12.50	-	-	-	1.00	5*	6	6	B1	0.23
**F14**	74.00	-	-	25.00	-	-	-	1.00	6	6	6	A	0.21
**F15**	49.00	-	-	50.00	-	-	-	1.00	6	6	6	A	0.18
**F16**	87.00	-	-	12.50	-	-	-	0.50	6	6	6	A	0.21
**F17**	74.50	-	-	25.00	-	-	-	0.50	6	6	6	A	0.19
**F18**	49.50	-	-	50.00	-	-	-	0.50	6	6	6	A	0.16
**F19**	87.40	-	-	12.50	-	-	-	0.10	5	5 *	5 *	B1	0.19
**F20**	74.90	-	-	25.00	-	-	-	0.10	6	6	6	A	0.17
**F21**	49.90	-	-	50.00	-	-	-	0.10	6	6	6	A	0.14
**F22**	87.45	-	-	12.50	-	-	-	0.05	4	4	4	A	0.19
**F23**	74.95	-	-	25.00	-	-	-	0.05	6	6	6*	B	0.17
**F24**	49.95	-	-	50.00	-	-	-	0.05	6	6	6	A	0.14
**F25**	-	86.50	-	12.50	-	-	-	1.00	6	6	6*	B	0.23
**F26**	-	74.00	-	25.00	-	-	-	1.00	6	6	6	A	0.21
**F27**	-	49.00	-	50.00	-	-	-	1.00	6	6	6	A	0.18
**F28**	-	87.00	-	12.50	-	-	-	0.50	6	6	6	A	0.20

**F29**	-	74.50	-	25.00	-	-	-	0.50	6	6*	6	B	0.19
**F30**	-	49.50	-	50.00	-	-	-	0.50	6	6	6	A	0.15
**F31**	-	87.40	-	12.50	-	-	-	0.10	6*	6	6*	B	0.19
**F32**	-	74.90	-	25.00	-	-	-	0.10	6	6	6	A	0.17
**F33**	-	49.90	-	50.00	-	-	-	0.10	6	6	6	A	0.14
**F34**	-	87.45	-	12.50	-	-	-	0.05	5	5 *	5 *	B1	0.18
**F35**	-	74.95	-	25.00	-	-	-	0.05	5 *	6	5 *	B1	0.17
**F36**	-	49.95	-	50.00	-	-	-	0.05	6	6	6	A	0.13
**F37**	-	-	86.50	12.50	-	-	-	1.00	6	6	6	A	0.23
**F38**	-	-	74.00	25.00	-	-	-	1.00	6	6	6	A	0.21
**F39**	-	-	49.00	50.00	-	-	-	1.00	6	6	6	A	0.18
**F40**	-	-	87.00	12.50	-	-	-	0.50	6	6	6	A	0.20
**F41**	-	-	74.50	25.00	-	-	-	0.50	6	6	6	A	0.19
**F42**	-	-	49.50	50.00	-	-	-	0.50	6	6	6	A	0.15
**F43**	-	-	87.40	12.50	-	-	-	0.10	6	6	6	A	0.19
**F44**	-	-	74.90	25.00	-	-	-	0.10	6	6	6	A	0.17
**F45**	-	-	49.90	50.00	-	-	-	0.10	6	6	6	A	0.14
**F46**	-	-	87.45	12.50	-	-	-	0.05	5 *	5 *	5 *	B2	0.18
**F47**	-	-	74.95	25.00	-	-	-	0.05	6	6	6	A	0.17
**F48**	-	-	49.95	50.00	-	-	-	0.05	6	6	6	A	0.13
**F49**	-	86.50	-	-	12.50	-	-	1.00	6	6	6	A	0.22
**F50**	-	74.00	-	-	25.00	-	-	1.00	6	6	6	A	0.20
**F51**	-	49.00	-	-	50.00	-	-	1.00	6	6	6	A	0.17
**F52**	-	87.00	-	-	12.50	-	-	0.50	6	6	6	A	0.20
**F53**	-	74.50	-	-	25.00	-	-	0.50	6	6	6	A	0.18
**F54**	-	49.50	-	-	50.00	-	-	0.50	6	6	6	A	0.14
**F55**	-	87.40	-	-	12.50	-	-	0.10	6 *	6 *	6 *	B	0.18
**F56**	-	74.90	-	-	25.00	-	-	0.10	6 *	6	6	B	0.16
**F57**	-	49.90	-	-	50.00	-	-	0.10	6 *	6	6	B	0.13
**F58**	-	87.45	-	-	12.50	-	-	0.05	4	4	5 *	B1	0.18
**F59**	-	74.95	-	-	25.00	-	-	0.05	4	4	5 *	B1	0.16
**F60**	-	49.95	-	-	50.00	-	-	0.05	4	5	5 *	B1	0.12
**F61**	-	-	86.50	-	12.50	-	-	1.00	6	6	6	A	0.22
**F62**	-	-	74.00	-	25.00	-	-	1.00	6	6	6	A	0.20

**F63**	-	-	49.00	-	50.00	-	-	1.00	6	6	6	A	0.16
**F64**	-	-	87.00	-	12.50	-	-	0.50	6	6	6	A	0.20
**F65**	-	-	74.50	-	25.00	-	-	0.50	6	6	6	A	0.18
**F66**	-	-	49.50	-	50.00	-	-	0.50	6	6	6	A	0.14
**F67**	-	-	87.40	-	12.50	-	-	0.10	6*	6	6	B	0.18
**F68**	-	-	74.90	-	25.00	-	-	0.10	6	6	6	A	0.16
**F69**	-	-	49.90	-	50.00	-	-	0.10	6	6	6	A	0.12
**F70**	-	-	87.45	-	12.50	-	-	0.05	5*	5*	5*	B2	0.18
**F71**	-	-	74.95	-	25.00	-	-	0.05	5*	5	5	B1	0.16
**F72**	-	-	49.95	-	50.00	-	-	0.05	5*	5	5	B1	0.12
**F73**	-	86.50	-	-	-	12.50	-	1.00	6	6	6	A	0.23
**F74**	-	74.00	-	-	-	25.00	-	1.00	6	6	6	A	0.21
**F75**	-	49.00	-	-	-	50.00	-	1.00	6	6	6	A	0.18
**F76**	-	87.00	-	-	-	12.50	-	0.50	6 *	6	6	B	0.21
**F77**	-	74.50	-	-	-	25.00	-	0.50	6	6	6	A	0.19
**F78**	-	49.50	-	-	-	50.00	-	0.50	6	6	6	A	0.16
**F79**	-	87.40	-	-	-	12.50	-	0.10	5 *	6	4	B1	0.19
**F80**	-	74.90	-	-	-	25.00	-	0.10	6 *	6	6	B	0.17
**F81**	-	49.90	-	-	-	50.00	-	0.10	6 *	6 *	6	B	0.14
**F82**	-	87.45	-	-	-	12.50	-	0.05	4	5 *	6	B1	0.19
**F83**	-	74.95	-	-	-	25.00	-	0.05	4	5 *	5 *	B1	0.17
**F84**	-	49.95	-	-	-	50.00	-	0.05	5 *	5 *	5 *	B2	0.14
**F85**	-	-	86.50	-	-	12.50	-	1.00	6	6	6	A	0.22
**F86**	-	-	74.00	-	-	25.00	-	1.00	6	6	6	A	0.21
**F87**	-	-	49.00	-	-	50.00	-	1.00	6	6	6	A	0.18
**F88**	-	-	87.00	-	-	12.50	-	0.50	6	6	6	A	0.20
**F89**	-	-	74.50	-	-	25.00	-	0.50	6	6	6	A	0.19
**F90**	-	-	49.50	-	-	50.00	-	0.50	6	6	6	A	0.16
**F91**	-	-	87.40	-	-	12.50	-	0.10	6*	6	6	B	0.18
**F92**	-	-	74.90	-	-	25.00	-	0.10	6	6	6	A	0.17
**F93**	-	-	49.90	-	-	50.00	-	0.10	6	6	6	A	0.14
**F94**	-	-	87.45	-	-	12.50	-	0.05	5*	6	6	B1	0.18
**F95**	-	-	74.95	-	-	25.00	-	0.05	5*	6	6	B1	0.17
**F96**	-	-	49.95	-	-	50.00	-	0.05	6	6	6	A	0.14

**F97**	-	-	94.00	-	-	-	5.00	1.00	6	6	6	A	0.23
**F98**	-	-	89.00	-	-	-	10.00	1.00	6	6	6	A	0.23
**F99**	-	-	79.00	-	-	-	20.00	1.00	6	6	6	A	0.22
**F100**	-	-	94.50	-	-	-	5.00	0.50	6	6	6	A	0.21
**F101**	-	-	89.50	-	-	-	10.00	0.50	6	6	6	A	0.21
**F102**	-	-	79.50	-	-	-	20.00	0.50	6	6	6	A	0.20
**F103**	-	-	94.90	-	-	-	5.00	0.10	6	6	6	A	0.19
**F104**	-	-	89.90	-	-	-	10.00	0.10	6	6	6	A	0.19
**F105**	-	-	79.90	-	-	-	20.00	0.10	6	6	6	A	0.18
**F106**	-	-	94.95	-	-	-	5.00	0.05	6	6	6	A	0.19
**F107**	-	-	89.95	-	-	-	10.00	0.05	6	6	6	A	0.19
**F108**	-	-	79.95	-	-	-	20.00	0.05	6	6	6	A	0.18
**F109**	-	94.00	-	-	-	-	5.00	1.00	6	6	6	A	0.24
**F110**	-	89.00	-	-	-	-	10.00	1.00	6	6	6	A	0.24
**F111**	-	79.00	-	-	-	-	20.00	1.00	6	6	6	A	0.23
**F112**	-	94.50	-	-	-	-	5.00	0.50	6	6	6	A	0.22
**F113**	-	89.50	-	-	-	-	10.00	0.50	6	6	6	A	0.21
**F114**	-	79.50	-	-	-	-	20.00	0.50	6	6 *	6	B	0.20
**F115**	-	94.90	-	-	-	-	5.00	0.10	5 *	6	6	B1	0.20
**F116**	-	89.90	-	-	-	-	10.00	0.10	6	6	6	A	0.20
**F117**	-	79.90	-	-	-	-	20.00	0.10	6	6	6	A	0.19
**F118**	-	94.95	-	-	-	-	5.00	0.05	5 *	6	5*	B1	0.20
**F119**	-	89.95	-	-	-	-	10.00	0.05	6 *	6	6	B	0.19
**F120**	-	79.95	-	-	-	-	20.00	0.05	6	5*	6	B1	0.18
**F121**	94.00	-	-	-	-	-	5.00	1.00	6	6	6	A	0.24
**F122**	89.00	-	-	-	-	-	10.00	1.00	6	6	6	A	0.24
**F123**	79.00	-	-	-	-	-	20.00	1.00	6	6	6	A	0.23
**F124**	94.50	-	-	-	-	-	5.00	0.50	6 *	6 *	6	B	0.22
**F125**	89.50	-	-	-	-	-	10.00	0.50	6	6	6	A	0.22
**F126**	79.50	-	-	-	-	-	20.00	0.50	6	6	6	A	0.21
**F127**	94.90	-	-	-	-	-	5.00	0.10	5 *	5	6 *	B1	0.20
**F128**	89.90	-	-	-	-	-	10.00	0.10	6	6	6	A	0.20
**F129**	79.90	-	-	-	-	-	20.00	0.10	6	6	6	A	0.19
**F130**	94.95	-	-	-	-	-	5.00	0.05	4	5 *	5 *	B1	0.20

**F131**	89.95	-	-	-	-	-	10.00	0.05	4	4	5 *	B1	0.20
**F132**	79.95	-	-	-	-	-	20.00	0.05	6	6	6	A	0.19
**F133**	86.50	-	-	-	-	12.50	-	1.00	5 *	5 *	4	B1	0.23
**F134**	74.00	-	-	-	-	25.00	-	1.00	6 *	6 *	6 *	B	0.22
**F135**	49.00	-	-	-	-	50.00	-	1.00	6 *	4	4	B	0.18
**F136**	87.00	-	-	-	-	12.50	-	0.50	5 *	5 *	5 *	B2	0.21
**F137**	74.50	-	-	-	-	25.00	-	0.50	5 *	6 *	6	B1	0.19
**F138**	49.50	-	-	-	-	50.00	-	0.50	5 *	6 *	6	B1	0.16
**F139**	87.40	-	-	-	-	12.50	-	0.10	5 *	5 *	5 *	B2	0.19
**F140**	74.90	-	-	-	-	25.00	-	0.10	4	5*	5 *	B1	0.18
**F141**	49.90	-	-	-	-	50.00	-	0.10	5 *	5 *	6 *	B1	0.14
**F142**	87.45	-	-	-	-	12.50	-	0.05	4	4	4	A	0.19
**F143**	74.95	-	-	-	-	25.00	-	0.05	4	4	5	B	0.17
**F144**	49.95	-	-	-	-	50.00	-	0.05	4	5	4	B	0.14
**F145**	86.50	-	-	-	12.50	-	-	1.00	5 *	6 *	6 *	B1	0.23
**F146**	74.00	-	-	-	25.00	-	-	1.00	6	6	6	A	0.21
**F147**	49.00	-	-	-	50.00	-	-	1.00	4	6	4	A	0.17
**F148**	87.00	-	-	-	12.50	-	-	0.50	5 *	5 *	6 *	B1	0.21
**F149**	74.50	-	-	-	25.00	-	-	0.50	6	6	6	A	0.19
**F150**	49.50	-	-	-	50.00	-	-	0.50	6	6	6	A	0.14
**F151**	87.40	-	-	-	12.50	-	-	0.10	5	5 *	5 *	B1	0.19
**F152**	74.90	-	-	-	25.00	-	-	0.10	6 *	4	6	B	0.17
**F153**	49.90	-	-	-	50.00	-	-	0.10	6	6	6	A	0.13
**F154**	87.45	-	-	-	12.50	-	-	0.05	4	4	4	A	0.19
**F155**	74.95	-	-	-	25.00	-	-	0.05	4	6	4	A	0.17
**F156**	49.95	-	-	-	50.00	-	-	0.05	4	5	5*	B1	0.12
